# Vacancy assisted He-interstitial clustering and their elemental interaction at fcc-bcc semicoherent metallic interface

**DOI:** 10.1038/s41598-018-22141-y

**Published:** 2018-03-01

**Authors:** Ujjal Saikia, Munima B. Sahariah, César González, Ravindra Pandey

**Affiliations:** 1 Institute of Advanced Study in Science and Technology, Guwahati, 781035 India; 2 Departamento de Física Teórica de la Materia Condensada and Condensed Matter Physics Center (IFIMAC), Facultad de Ciencias, Universidad Autónoma de Madrid, E-28049 Madrid, Spain; 3 Department of Physics, Michigan Technological University, Houghton, MI 49931-1295 USA

## Abstract

Cu-Nb layered nanocomposite system can be considered as a prototype system to investigate stability of the fcc-bcc semicoherent metallic interfaces. Theoretical simulations based on density functional theory have been performed in order to investigate the stability of different defects in such interfaces. The calculations find the interfacial misfit dislocation intersections as the preferred site for defects including a vacancy, He-interstitial, and a vacancy-He complex in good agreement with previous works. Our results suggest that the presence of a metallic vacancy may act as a sink for defect and favour the migration of He interstitials leading to their aggregation at the interface. The potential capability of the vacancy to accommodate He atoms was also predicted with a higher affinity towards Nb. This aggregation of He atoms is driven by local density of electron and strain in a region in the neighbourhood of Nb. Finally, we propose a plausible picture of defect energetics in the vicinity of the interface based on the Voronoi volume and Bader’s charge analysis. This analysis may replace the conventional methods used for surface energetics mapping which are extremely tedious for such large systems.

## Introduction

The face-centered cubic (fcc) and the body-centered cubic (bcc) semicoherent layered metallic nanocomposite systems have engrossed as a viable structural material for the next generation of nuclear reactors^1–3^. Enhanced mechanical stability, ability to withstand a very high temperature environment and self annihilation of radiation induced defects make them suitable candidates in other extreme environment applications as well^4–8^. The easy accessibility for the defects and a favorable environment for recombination of Frenkel pairs suggest the incorporation of materials with a large interfacial region where the radiation induced defects annihilation could be facilitated^2,9,10^. It is to be noted that applications for extreme environment require the materials properties to be primarily governed by the structure and chemistry of the interface. The complexity of the fcc-bcc interface structure is the key factor here and it has been theoretically confirmed that a tailored interface can substantially enhance these properties^2^. The exotic nature of the interface is attributed to different crystal symmetry and bonding characteristics of the participating materials.

Detailed knowledge about the effect and crucial role of defects on the performance of the material is very essential before engineering them for the extreme environment applications. Materials used in nuclear fusion reactors will be always exposed to very high doses of radiation which may lead to a large number of defects such as vacancies and interstitials^11,12^. Interstitial like He may accumulate inside the material causing damage by formation of voids^13^. Additionally, the presence of vacancy has an enormous impact on clustering, segregation and precipitation of the solute atoms^14–17^. Solute diffusion mechanism is controlled by the strength of interaction between a vacancy and the solute atom, which can vary for different types of elements. Therefore, understanding the elemental process of vacancy-interstitial atom interaction in fcc-bcc semicoherent interfaces is of particular interest in this study.

Density functional theory (DFT) calculations offer very useful and reliable information on material properties at 0 K temperature. Such information can serve as input for large scale calculations like molecular dynamics (MD) and kinetic Monte Carlo (kMC) simulations at nonzero temperatures^18,19^. Techniques like *ab initio* parametrized kinetic Monte Carlo has already shown great potential to treat the microstructural evolution of materials in the condition of radiation damage/He production and impurity diffusion in realistic and rough grain boundary structures^20,21^. Demkowicz’s group constructed different DFT-based embedded atom method (EAM) potentials in order to perform extensive MD calculations by considering Cu-Nb as a prototype fcc-bcc nanocomposite system. They studied the interface energetics, structure, mechanical properties as well as clustering and migration behavior of vacancies or He interstitials^22–26^. For the layered Cu-Nb system, both experimental and theoretical studies predicted the Kurdjumov-Sachs (KS) orientation as the preferred interfacial orientation^27,28^.

The necessity of a large simulation cell to capture the topology of the non-coherent interface and strain field generated near the interface was the major challenge to perform DFT based calculation on Cu-Nb layered system, which was nicely untangled by Metasanurk *et al*. with the introduction of a quasi unit cell of reasonable size^29^. They performed first-principles simulations on the metallic vacancy and self-interstitial formation energy in the Cu-Nb system, showing the most stable sites at the misfit dislocation intersection (MDI), *i*.*e*. Cu/Nb atom sites at the top of the other species. Another subsequent work reveals that the vacancy clusters are stable up to four vacancies and the migration of monovacancy between two neighboring MDI’s is unlikely due to the high energy barriers^30^. González *et al*.^31^ expanded the analysis to the He interstitial atoms and the He-vacancy complex in the vicinity of the Cu-Nb semicoherent interface in the KS orientation. In that work, the prominent trapping exerted by the Cu-Nb interface on metallic vacancies and He interstitial atoms were studied with the help of metallic vacancy/He interstitial formation energy and DFT-based defect migration barrier calculations.

In this work, we have performed first-principles DFT calculations to explore the vacancy-interstitial elemental interaction mechanism in Cu-Nb layered nanocomposite system. We have shown that a unit cell consisting of four layers of Cu and four layers of Nb is sufficient to capture the strain field generated near the interface. Here, our study extends the analysis to formation energies of different types of point defects at the MDI and NON-MDI regions of the interfacial Cu and Nb layers. Moreover, considering their important contribution in processes like migration and clustering of defects in the host matrix, defect-defect and host-defect interactions were also studied in detail. The results of our study agree well with the previous literature^29,31^ in predicting MDI regions to be the most stable sites for monovacancy and isolated He interstitials. Considering the importance of He trapping on the performance of materials properties, the formation of metallic monovacancy and He atom complexes (up to 5He atoms) at the MDI region of the interfacial layers were also studied. To shed light on interaction between a vacancy and He interstitial atom, we have calculated the formation and trapping energy for each He interstitial atom in these complex at the MDI region of the interfacial Cu and Nb layers. Charge density difference and electronic density of states (DOS) analysis were performed to understand the ongoing interaction between metallic vacancy and He interstitial atoms. Finally, inspired by the study performed by Choudhury *et al*.^32^, we have performed Voronoi volume and Bader charge analysis to understand the point defect energetics at the interface of the fcc-bcc semicoherent metallic system.

## Results

### Formation energy of the defects on different interfacial sites

The first step in our procedure consists of the validation of the computational DFT parameters in order to show that the obtained results are not artifacts of the restrictions implemented in our simulations. Therefore we have tested the size of the supercell, energy cutoff for plane wave and the number of k points used in our DFT calculations. We found that our 4-layered structure with Γ-point, which is computationally less expensive in comparison to the 6-layered system, is sufficient to replicate the physics of the Cu-Nb layered semicoherent interface (more information is provided in the supporting information (SI)).

**Figure 1:**
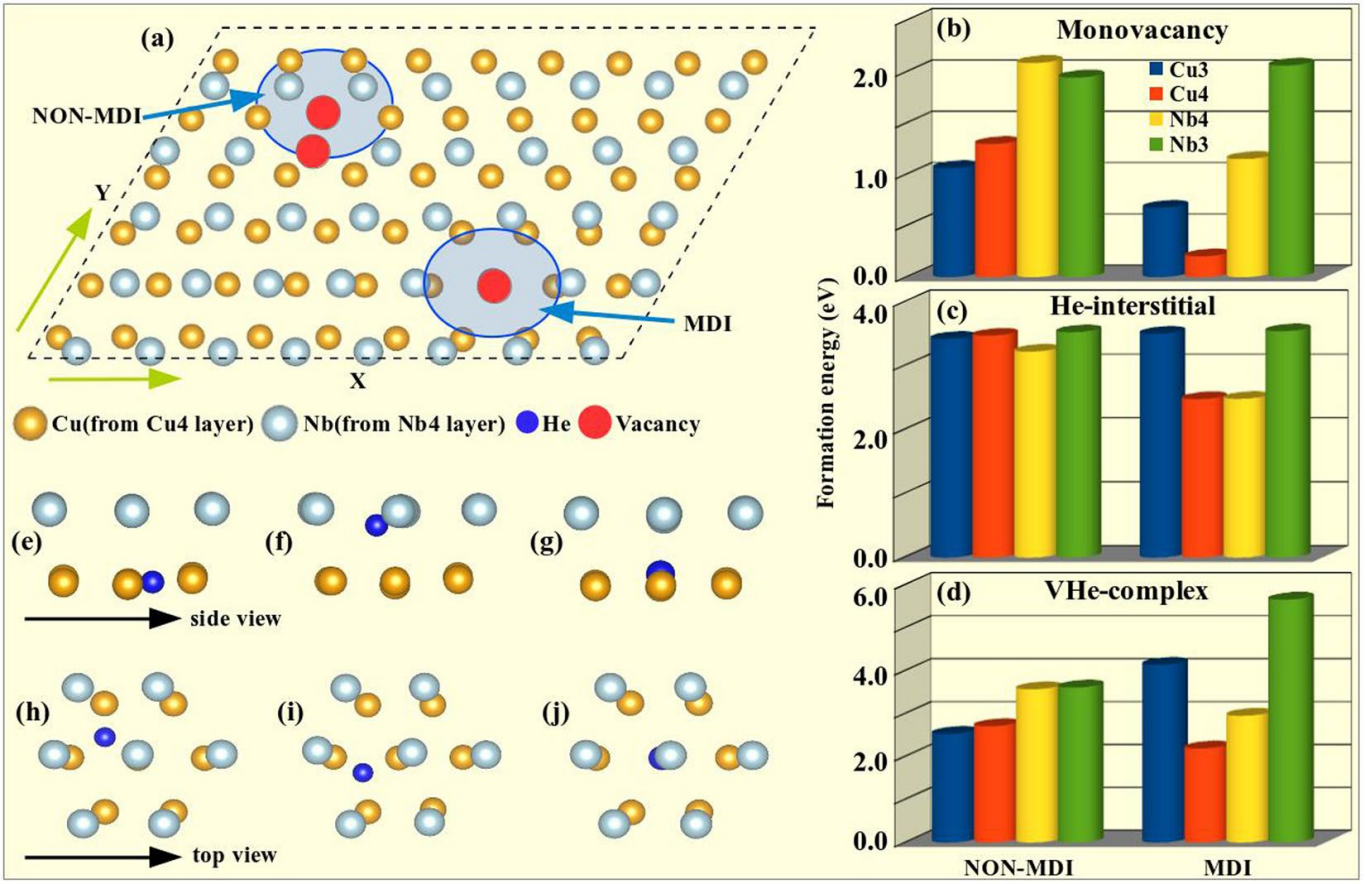
(**a**) Top view of the first neighbouring interfacial (Cu4-Nb4) layers. Red dots inside the blue circles represent the selected site for a Cu or Nb vacancy in the first neighbouring interfacial layers. At the MDI region, Cu and Nb vacancy position overlaps with each other. Formation energy of V, He and VHe complex at MDI and NON-MDI sites of the first and second neighbouring interfacial layers is shown in figure (**b**–**d**) respectively. (**e**) The unrelaxed configuration of a He atom at the MDI region of the Cu4 layer. (**f**) After structural relaxation the He atom finds its stable position near the Nb4 layer, (**g**) but insertion of a metallic vacancy at the MDI region of the Cu4 layer makes the He atom to come back to the Cu4 layer and occupy the metallic vacancy position. The top view of the same configuration as shown in (**e**–**g**) is depicted in (**h**–**j**) respectively.

Previous studies identified that the interfacial MDIs are the most stable areas for point defects in the Cu-Nb layered system. To reaffirm this fact, we have considered two different areas in each first neighbouring interfacial layers Cu4 and Nb4 (see Fig. 1(a) and the whole unit cell in SI Fig. SI1), namely, MDI and NON-MDI (where there is no lattice point matching across the interface) as shown in Fig. 1(a). In order to explore the effect of interface on defect formation energy, we have extended the V, He and VHe complex formation energy calculation to the second neighbouring interfacial layers (Cu3 and Nb3) as well. For Cu4 and Nb4 layers, V, He and VHe complex formation energy trends are consistent with the previous study (*i*.*e*., formation energy at MDI is lower than at NON-MDI sites, Fig. 1(b–d)). But the situation changes as we deviate from the interface. For Cu3 layer, MDI region remains favourable site for monovacancy, having a lower value of 0.68 *eV* (1.06 *eV* at NON-MDI). But for Nb3 layer, the value at MDI (2.06 *eV*) and NON-MDI (1.95 *eV*) region is almost comparable. This can be explained through the strain energy associated with interfacial and nearby atoms due to lattice mismatch across the fcc-bcc semicoherent interface considered in this study. For Cu, the effect of this strain field is extended to the second neighboring layer. Hence, contrary to the Nb3 layer, the Cu3 layer at the MDI region has less monovacancy formation energy.

On the other hand, He-interstitial finds enough free space to accommodate at interfacial MDI, and that is the reason why formation energy (2.48 *eV*) for both layers is less than at NON-MDI region (3.48 *eV* and 3.22 *eV* for Cu4 and Nb4 respectively), as explained before^31^. But if we insert a He atom in the Cu3 or Nb3 layers, because of the similar lattice environment, the available space for the interstitial He atom will be almost same for both MDI (3.50 *eV* at Cu3 and 3.54 *eV* Nb3) and NON-MDI (3.42 *eV* at Cu3 and 3.52 *eV* Nb3) region of that particular layer. As a result, the formation energy will also become the same (Fig. 1(c)). The He atoms can emerge at the NON-MDI areas and probably they will fall to lower energy positions close to the MDI areas.

For VHe complex, the scenario changes. For Cu3 and Nb3 layers formation energy at MDI (4.14 *eV* for Cu3 and 5.65 *eV* for Nb3) is much larger than at NON-MDI areas (2.53 *eV* for Cu3 and 3.61 *eV* for Nb3). At the interfacial MDI region, Cu atoms fall nearly on the top of a Nb atom which makes this region highly strained. Removal of a Cu (Nb) atom from the MDI region of the Cu4 (Nb4) layer is accompanied with release of large amount of strain energy which makes insertion of He-interstitial atom less expensive energetically. On the contrary, when a He atom occupies a vacancy at the Cu3 or Nb3 layers, more energy is required at the MDI than at the NON-MDI region at both sides (Fig. 1(d)). In this case, although we created a vacancy in the MDI region of the Cu3 or Nb3 layer, we kept the highly aligned metallic atoms in the MDI region of the first neighbouring interfacial layers. In such case, insertion of a He-interstitial at the MDI region of Cu3 or Nb3 layer will increase the strain, which is energetically unfavourable and renders a lower VHe complex formation energy at the NON-MDI regions.

Another interesting fact we observed is the affinity of the interstitial He atoms towards Nb. When a He atom is placed in the Nb4 layer it stays close to that layer. On the other hand, for an initial position close to the Cu4 layer (see Fig. 1(e,h)), it also always prefers to find a stable position near the Nb4 layer after relaxation (see Fig. 1(f,i)). In the second step, we inserted a Cu vacancy at the MDI site of the Cu4 layer and re-relaxed the structure. Interestingly, the He interstitial comes back to the Cu4 layer and occupies the Cu vacancy position. Such behavior of He interstitial in the interfacial region suggests that although He interstitial has the higher affinity towards Nb, presence of a metallic vacancy in the Cu layer may capture the He interstitial around it. From Fig. 1(f), we noticed that the He atom induces small deformation in the neighbouring Nb atoms, which was restricted along the Nb atomic plane and decreases after insertion of the metallic vacancy 0(g). The average change observed in X and Y position of the neighbouring Nb atoms with respect to the initial system was 0.31 Å and 0.13 Å which reduces to 0.13 Å and 0.03 Å in presence of the vacancy. Hence, this vacancy may act as a sink for defect capturing and influence the migration of interstitials which on the other hand plays a crucial role in clustering of interstitials. This is not a surprise, the He atoms tends to occupy the empty spaces reducing the repulsion with the metallic atoms around as it was previously proposed for bcc metals^33^.

**Figure 2:**
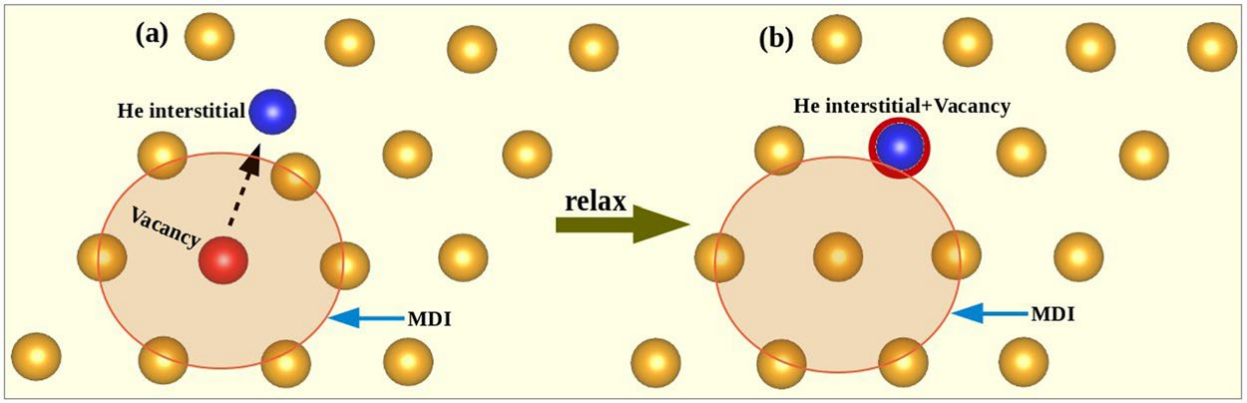
(**a**) The unrelaxed configuration of a Cu-monovacancy at the MDI region and a He interstitial atom away from the MDI region of the interfacial Cu layer. (**b**) After structural relaxation the Cu-monovacancy moves towards the He interstitial and recombination occurs between them.

In this section we have seen the most preferred site is at MDI areas for a Cu or Nb monovacancy and an interstitial He atom in between the first neighbouring interfacial layers always move towards the nearby metallic vacancy site and occupies the vacancy position at the regular lattice site. Such behavior of He interstitial located in between two interfacial layers suggest that they are more mobile than the metallic monovacancy at the regular lattice site of the interfacial layers. Motivated by this fact, we tried to explore the situation when the He interstitial is located in the same plane where a metallic Cu-monovacancy is situated in the MDI site of the interfacial layer as shown in Fig. 2(a). Interestingly, after structural relaxation, the He interstitial didn’t move towards the metallic monovacancy site directly this time. Rather, the metallic monovacancy moved towards the lattice site nearest to the He interstitial where it recombined with the He interstitial (Fig. 2(b)). This result indicates that within a particular atomic layer, metallic monovacancies are more mobile than the interstitials which forced the metallic vacancies to move even from its most stable site (the MDI site). This means that the energy cost for the He atom to find the vacancy is larger than the movement of the metallic atom to cover the hole leaving a vacancy behind (finally occupied by the He atom). The VHe configuration shown in Fig. 2(b) has formation energy of 2.19 eV, which is 0.21 eV larger than the formation energy of the same defect configuration at the MDI region. Such procedure will allow the accumulation of vacancy or VHe complexes around the MDI.

### Interaction between defects

Although a few semi-empirical approaches have already been carried out to study the He interstitial clustering at the Cu-Nb interfaces^34,35^, there are no DFT results reported so far. As discussed in the previous section, the most energetically preferred site found for a He interstitial is the MDI region of the interface. In the first step of our analysis, we have followed the results and structures presented in reference^31^. In this work, C. González *et al*. confirmed that interfacial misfit dislocation intersection (MDI) regions are the most favourable area for a metallic monovacancy (V), a He interstitial and a V + 1He complex. Therefore we have focused our attention to the energetic study of the He-clusters at the MDI regions where they can be formed more easily. We have inserted the He atoms randomly near the area of interest (*i*.*e*., the MDI region) and the system was relaxed until it found the optimal position for each He atom. When we included more atoms close to the initial He atom, we have followed symmetric geometrical configurations: lines, triangles, squares or tetrahedral structures. This has been performed for both initial and defective (metallic monovacancy at the MDI site of the Cu4/Nb4 layer) interface. To ensure that the final structure is not stuck in a local minimum, we tried with different configurations of the He atoms with initial positions close to the area of interest and found that those configurations which converge to the final structure considered here are lower in energy than the others. However, one should insert the He atoms not far from the area of interest to prevent the final structure from getting stuck into some local minimum. To give a check, we have considered two He atoms, when we put them apart (one at MDI and one at NON-MDI) the formation energy increases (for the initial/defective interface, 5.94 eV/5.40 eV on Cu4 and 5.80 eV/6.22 eV on Nb4 layer) than two He atoms put together in the MDI region (for the initial/defective interface, 5.29 eV/4.28 eV on Cu4 and 5.28 eV/4.42 eV on Nb4 layer). These results can help in the development of new better Cu-He-Nb potentials for MD.

For the initial interface, both the interfacial layers expel the inserted He interstitials to the interface region. As happened with a single He atom, they always found stable accommodation near the Nb4 layer (the energetically most favorable structures are provided in the SI, Fig. SI4). For the defective interface, a single He atom always prefers to stay at the vacancy center in both interfacial layers. The second He atom inserted at the MDI region of the Cu4 layer budge towards the Nb4 layer. The same case happens with the third, fourth and fifth He atoms (the energetically most favorable structures are provided in the SI, Fig. SI5). Interestingly, unlike Cu-monovacancy, a single Nb-vacancy at the MDI site of the Nb4 layer accommodates up to four He atoms. The fifth He atom also sticks near the Nb-vacancy but slightly gets out of the Nb4 atomic layer. Presence of metallic vacancy influences the arrangement of the He atoms considerably and reduces the structural deformation occurred in the metallic matrix. We measured this deformation in terms of change in dimension of the MDI region (Δ*x* and Δ*y* values in the SI Figs SI4 and SI5).

**Figure 3:**
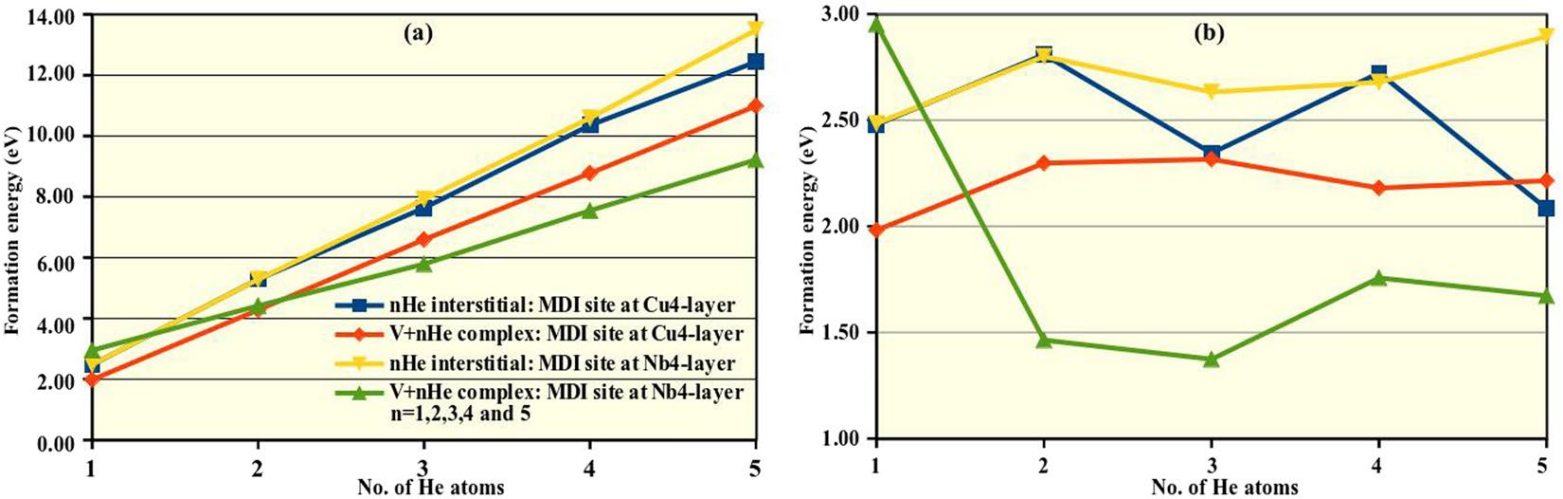
(**a**) Formation energy of nHe (n = 1, 2, 3, 4 and 5) interstitial clusters at the MDI region of the interfacial layers without (blue and yellow) and with (red and green) a metallic monovacancy at the respective MDI region. (**b**) Formation energy of the added He atom to the (n−1)He complexes for the same configurations. Line colours and symbols has same meaning as in figure (a).

The formation energy of nHe interstitials at the MDI region with and without a metallic monovacancy can be compared to their corresponding bulk values available in the literature^36,37^. We observed that the formation energy values for different defect configurations are significantly lower than in their corresponding bulk counterparts, thus justifying the interface as a preferred area (data available in SI Table SI3). For both (Cu and Nb) the bulk metallic systems, the formation energy of the defect complexes increases as we increase the number of He (SI Fig. SI6). The presence of metallic vacancy lowers the formation energies in bulk Cu. Interestingly, presence of vacancy enhance the formation energy of the nHe complex in bulk Nb. This difference reduces as we increase the number of He atom and become almost equal for V + 4He complex. For the layered system, as shown in Fig. 3(a), the formation energy of the defect clusters increases with the increasing number of He for both cases (*i*.*e*., with and without a metallic monovacancy). For Cu-layer, the formation energy of the nHe complexes are less for all concentrations of He when there is a monovacancy sitting at the MDI site than without it. As for Nb-layer, the trend is little different. For a single He interstitial, larger formation energy is seen in presence of the metallic monovacancy. With increasing number of interstitials, change in formation energy is more rapid without the monovacancy. So, for higher complexes the formation energy trend become similar to that of the Cu-layer. Notably, the slope of the nHe formation energy curve in presence of a metallic monovacancy at the MDI site is lower than without the metallic monovacancy for both layers. This suggests that the presence of metallic vacancy may favour the He interstitial clustering, which is more eminent for interfacial Nb layer as we increase the number of interstitial He atoms.

The picture becomes more clear when we plotted the formation energy of the individual He atoms as they are getting added to the complex. As shown in Fig. 3(b), without a metallic monovacancy at the MDI site of the interfacial layers, the formation energy for the added He atom increases when we insert the second He atom. From the third He atom small fluctuation in formation energy trend has been observed. For Cu4 layer, similar trend was observed with comparably lower values of formation energy in presence of the metallic vacancy. This fluctuation arises because some He atoms find more stable accommodations near the Nb4 layer, thus lowering the formation energy. Interestingly, in presence of metallic monovacancy at the MDI site of the Nb4 layer, formation energy per He decreases gradually as we vary the number of He interstitial from 1 to 3 and remains smaller thereafter. Such observation also provides further evidence that presence of metallic vacancy favours the He interstitial clustering at the MDI site of the Nb4 layer.

**Figure 4:**
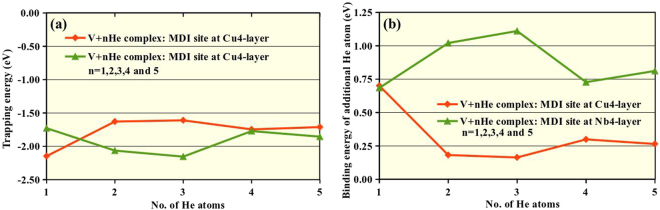
(**a**) Trapping and (**b**) binding energy for each He interstitial atom in the V + nHe (n = 1, 2, 3, 4 and 5) complexes at the MDI region of the interfacial Cu and Nb layers.

Further, to understand the He trapping mechanism in detail, we calculated the trapping energy for each He interstitial atom in the V + nHe (n = 1, 2, 3, 4 and 5) complexes using eq. 2. We defined the trapping energy *E*^*trap*^ to characterize the energy required for moving a He atom from a distant interstitial site into the metallic monovacancy at the MDI site of the first neighbouring interfacial layer. We choose distant interstitial site for He-interstitial in the Cu2 (Nb2) layer at a distance >10 Å from the metallic monovacancy at the MDI site of the Cu4 (Nb4) layer. By definition, a negative trapping energy indicates an exothermic process when the He atom moves from a distant interstitial site to the trapped vacancy site. Figure 4(a) shows that trapping a single He atom in a Cu-monovacancy site at the interfacial MDI region is most favourable having the lowest trapping energy of −2.15 eV. Inclusion of second He atom in the Cu-monovacancy site makes the trapping less favorable. The trapping remains the same as we increase the number of He atoms from two to three and again slightly goes down with the inclusion of the fourth and the fifth He atoms. On the contrary, inclusion of He atoms in the Nb-monovacancy at the MDI region of the interfacial Nb layer shows unmatching trend. Trapping a single He atom is least favourable having an energy of −1.73 eV. The trapping become more favourable with the inclusion of He atoms, showing the most favourable energy for three He atoms in the single Nb-vacancy site with a value of −2.15 eV, similar to the trapping energy of a single He atom in the Cu-monovacancy site. With the inclusion of fourth and fifth He atoms in the Nb-vacancy site, the trapping energy slightly goes up, indicating the less favourable situation. With four He atoms, the trapping energy in Cu and Nb layers matches with each other, probably because the He are close to Nb layer in both cases.

Such uncorrelated trapping behaviour for He atoms in Cu and Nb-monovacancy region can be explained from the binding energy. Figure 4(b) we have plotted the binding energy of an additional interstitial He atom binding to a V + (n−1)He complex to form a V + nHe complex as a function of n, using eq. 4. Our definition of binding energy is consistent with the one reported by M. A. Tschopp *et al*.^38^. Here, we have considered the V + nHe complexes at the MDI region of the first neighbouring interfacial layers. For He atoms in the Cu-vacancy site (orange line) the binding energy between a He atom and the V + (n−1)He complex decreases as the number of He atoms increases. As a result, the trapping exerted by the Cu-vacancy on the He atom also decreases. Attentive analysis of the optimized structures of the Cu-vacancy + nHe complexes also supports that only one He atom stays in the Cu-vacancy site, the He atoms inserted further (from 2nd to 5th He atoms) always prefer to go to the adjacent space between interfacial Cu and Nb layers (SI, Fig. SI5(g–j)). The binding energy curve for Nb-vacancy + nHe complexes (green line in Fig. 4(b)) shows that the binding between a He atom and V + (n−1)He complex increases as the number of He atoms increases, hence, the trapping energy is also increasing. For the fourth and fifth He atom in the Nb-vacancy site, due to limited space, the He atoms slightly goes away from the Nb-vacancy position (SI, Fig. SI5(t)), with reduced binding energy. Consequently, trapping also becomes less favourable for these configurations.

In conclusion, the inclusion of He atom into the Cu/Nb-vacancy site was exothermic (at least) till the fifth He atom even though we observe less favourable trapping after the inclusion of the second (fourth) He atom into the Cu (Nb) vacancy site. Such result indicates the potential capability of these vacancy sites to accommodate more He interstitials, and the inclusion process will be still energetically favourable. It would be a very interesting investigation to find the maximum number of He atoms one can add exothermically in these metallic vacancy sites. Unfortunately, due to limited computational resources, the performance of this study is highly inaccessible with DFT methodology.

### The mechanism of He-trapping

Within the electronic environment of the considered fcc-bcc semicoherent metallic matrix, several questions arise like, why He atoms show affinity towards interfacial Nb layer, why a metallic vacancy traps the He atoms or why presence of one He atom favors accommodation of the other He atoms. The first two questions have been simply answered before^31,33^. In the first case, the lower formation energy at the Nb bulk has been proposed as an explanation for the He attraction to the corresponding size at the interface. On the other hand, the He atoms prefers the empty areas where the repulsion of the metallic atoms can be reduced. Now these simple justifications are explained in terms of a charge density analysis. For that purpose, we have calculated the charge density difference and total/partial density of states (DOS) for the Cu-Nb layered system containing different V + nHe (n = 1 to 5) complexes.

In metals, bands of allowed electron states are filled up to the Fermi energy (*E*_*F*_). In case of Cu-Nb layered system, we have constructed the semicoherent metallic system by joining two metallic slabs having different Fermi levels (with a higher value in the Nb slab). Hence, upon formation of the Cu-Nb layered system, charge transfer will take place from the Nb slab to the Cu slab, to equalize the *E*_*F*_ of the combined system. The charge transferred from Nb to Cu slab has already been confirmed by a previous study^39^. As a result, a charge depleted (accumulated) region near the interfacial Nb (Cu) layer was observed. On the other hand, He atoms have a closed shell electronic structure and the energy consumption will be more in case of larger polarization of charge densities. As a result, He would always prefer to go to a low electron density region where the repulsion can be minimized. Such behaviour of He atoms inside a metallic system was nicely explained in the electophobic interaction model by Zhou *et al*.^40^. This explains the affinity of the He atoms towards the interfacial Nb layer. By creating a monovacancy at the MDI site of the interfacial Cu layer, a region with lower electron density is created. As a consequence, interstitial He atoms get trapped inside the metallic vacancy center, which explains the trapping of He at the metallic vacancy sites.

**Figure 5:**
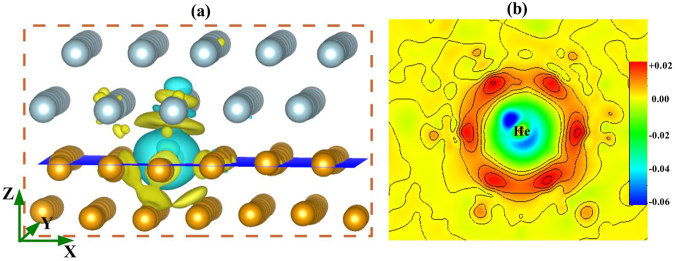
(**a**) 3-dimensional charge density difference plot with iso-surface value 0.005 e/Å^3^. Yellow and cyan regions show charge accumulation and depletion regions respectively. (**b**) 2-dimensional charge density difference plot along the X-Y plane (blue plane in the 3-D image) is plotted considering the net charge distribution along the Z-direction bounded by the orange dotted box in the 3-D image. For visual clarity, the region around the He atom is only depicted. The color scale is in e/Å^3^.

Then, we are interested to see what happens to the local charge distribution after insertion of a He atom at the metallic vacancy site. For that purpose, the charge density difference (Δ*ρ* = *ρ*_(*CuNb*,*V*+*He*)_ − *ρ*_(*CuNb*,*V*)_ − *ρ*_(*He*)_) has been plotted for a system with a metallic vacancy and a He atom at the MDI site of the Cu4 layer (Fig. 5). The presence of He atom at the metallic vacancy centre of the MDI region of the Cu4 layer instigates perturbation of the local charge density distributions (Fig. 5(a)), forming some charge depleted (cyan) and accumulated (yellow) regions around it. For clarity, we have plotted 2-dimensional projection of the net charge density difference for the region bounded by the orange dotted box along the X-Y plane (blue plane in the 3-D image) as shown in Fig. 5(b). This plot suggests that the He atom induces a low electron density region (blue and green region) around it surrounded by a electron accumulated region (red region). Hence, the presence of He atom creates a low electron density region around it which favours the accommodation of the next He atom in that region. At the same time, insertion of the second He atom in the vicinity of the first He atom will reduce the space available for them. As a result strain on the He atoms will increase, leading to an atomic rearrangement and a possible deformation of the surrounding area. In fact, such deformation was observed in the defective systems (see SI, Figs SI4 and SI5).

In support of this argument, we have calculated the average Voronoi volume (which is the measure of the free volume available for an atom) of the He atoms in the V + nHe (n = 1, 2, 3, 4 and 5) complexes (Table 1). Voronoi volume is inversely proportional to strain. Average Voronoi volume decreases with number of He atom at the V + nHe complex for both cases, indicating the increase in strain on the He atoms. Although the second He atom find low electron density region near the Cu-vacancy center, strain on the He atom influence it to move towards the Nb4 layer, where, it finds another low electron density region with more space to be accommodated. Same thing happens with the third, fourth and fifth He atom. On the other hand, He atoms find more space to accommodate near the Nb-vacancy center. That is why we have observed up to five He atom sticking to a single Nb-vacancy site. In summary, although we are unable to know the exact lowest volume requirement for accommodation of a He atom in the Cu-Nb layered system, based on the observation we have made in this section, we may say that, having at least ~9 Å^3^ of available volume in a low electron density region, there is a good chance for a stable accommodation area for a He atom. Hence, the charge density and available Voronoi volume of the region plays an important role in clustering of He in that region of the Cu-Nb layered system.

**Table 1:** Average Voronoi volume per He atoms in the V + nHe (n = 1, 2, 3, 4 and 5) complexes in Å^3^.

Configuration	MDI: Cu4	MDI: Nb4
V + 1He	12.19	19.24
V + 2He	11.38	13.96
V + 3He	10.00	11.78
V + 4He	9.44	10.61
V + 5He	9.16	9.88

**Figure 6:**
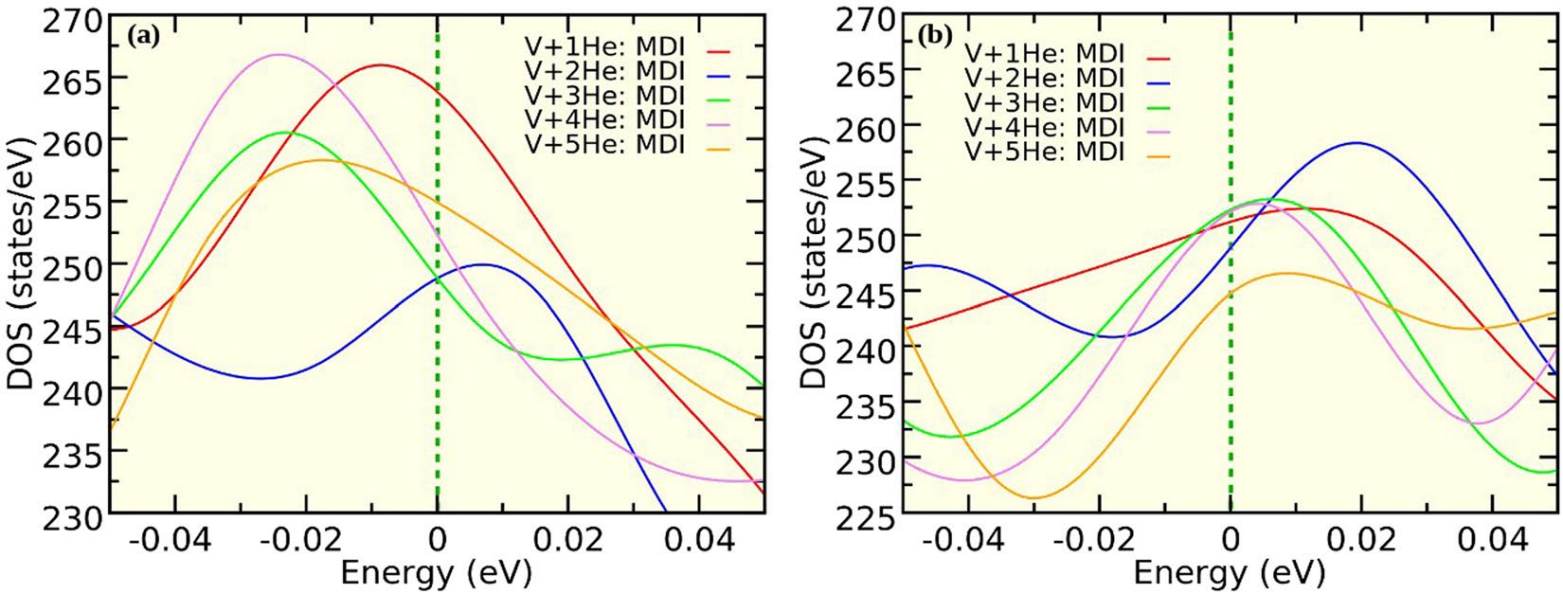
Total DOS for the systems containing V + nHe (n = 1, 2, 3, 4 and 5) complexes at the MDI region of the interfacial (**a**) Cu and (**b**) Nb layers. Fermi energy is set to zero.

**Figure 7:**
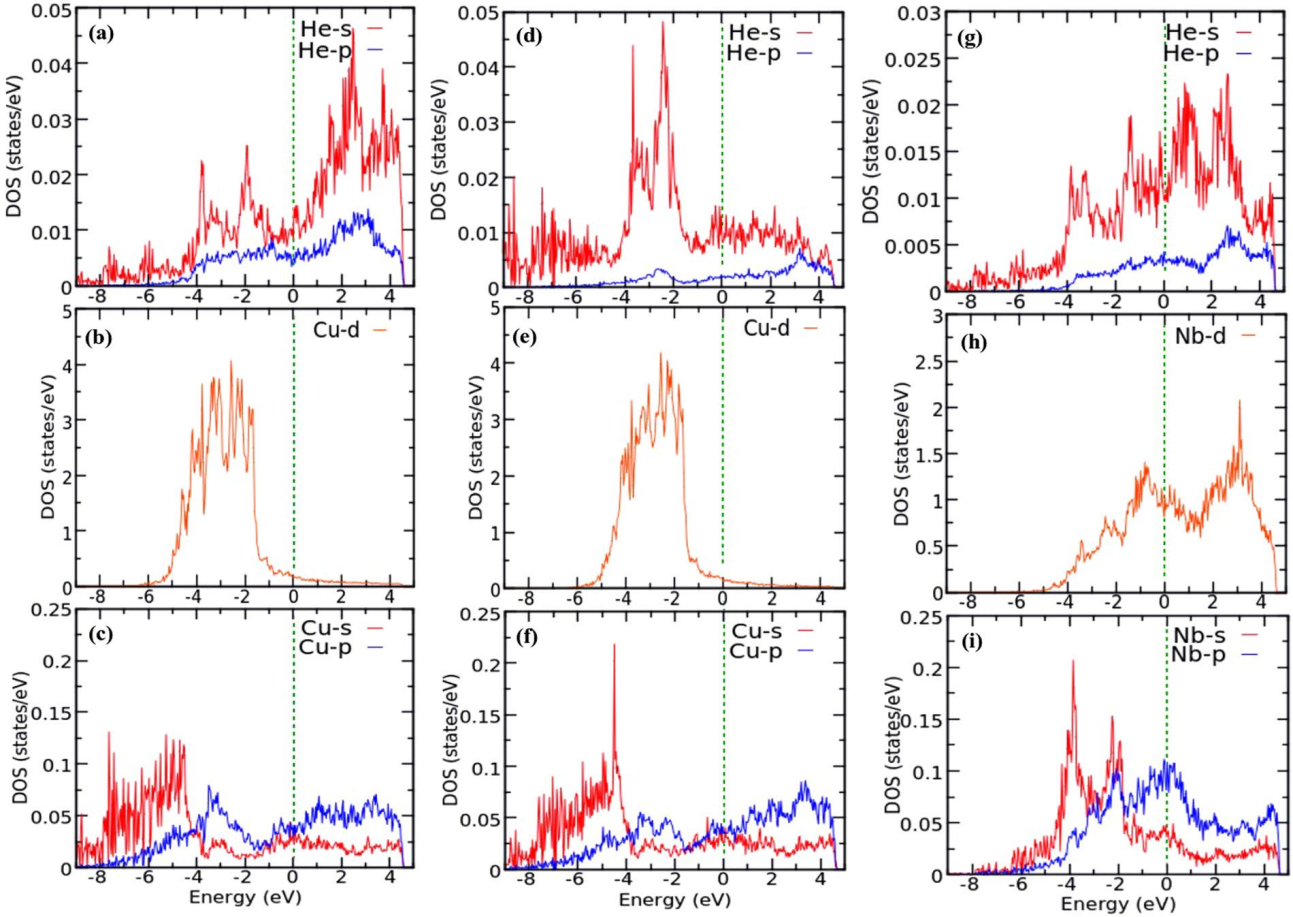
Orbital projected density of states (DOS) for He-s,p and metallic-s,p,d states. For the system with a He atom at the MDI region of the interfacial Cu layer the (**a**) He-s,p (**b**) Cu-d and (**c**) Cu-s,p states are shown (we consider a Cu atom nearest to the He). The same is depicted for a system with a He atom and Cu-monovacancy at the MDI site of the interfacial Cu layer (**d**–**f**), and a He atom and Nb-monovacancy at the MDI site of the interfacial Nb layer (**g**–**i**). Fermi energy is set to zero.

We have also calculated the total DOS for the defect complexes (V + nHe) at the interfacial Cu and Nb layers and plotted them in Fig. 6(a,b) respectively. As explained previously^41^, higher values of total DOS at the Fermi level represents energetically less favorable system. For the V + nHe complexes at the Cu4 layer, lowest value of DOS at the Fermi level is observed for the V + 2He and V + 3He complexes, implying the highest stability of the system with two He atoms in the vicinity of the Cu-monovacancy site. Inclusion of 4th and 5th He atom near the Cu-monovacancy site makes the system energetically less favorable. On the contrary, as shown in Fig. 6(b), the system with 5He atoms inside a Nb-vacancy shows the highest stability, having the lowest DOS at the Fermi level. We have also calculated the orbital projected DOS for the system with a He atom close to the Cu4 layer at the MDI area with and without a Cu-monovacany. Hybridization between He-*s* and Cu-*d* states were observed in presence of metallic vacancy (Fig. 7(d–f)). Similarly, when we have considered a He atom within a Nb-monovacancy, an influence on the DOS of He was observed (Fig. 7(g–i)). Such change may imply chemical interaction between He and metallic atoms. To shed light on this point, we have performed Bader charge analysis on the Cu-Nb layered system having V + nHe complexes in it. Our analysis confirms charge transfer from the neighbouring metal atoms to the He atoms (SI, Table SI4), suggesting any chemical interaction between the He atom and the surrounding metallic atoms.

### Surface energetics mapping with Bader charge and Voronoi volume

As already discussed in the introduction section, an extensive energetic study based on DFT-methodology was previously performed on point defect energetics for a Cu-Nb metallic layered system^31^, building an energetic map from the calculations of He interstitial atoms as well as both metallic vacancies at different points of the interface. In this section, rather than such laborious and computationally expensive DFT-calculations, we have realized the behaviour of point defects at the interface of the Cu-Nb layered system using Voronoi volume and Bader charge. Our previous results suggested the tendency of charge accumulation (depletion) of the interfacial Cu (Nb) atoms^39^. Based on this idea, we have analysed the change in Bader charge (number of electrons on the interfacial Cu and Nb atoms compared to their bulk counterpart) of the interfacial Cu and Nb atoms, which could be the signature of the chemically favorable regions for accommodation of interstitial atoms having closed shell electronic structure like He. At the same time, the increase in strain energy of the system was also realized in terms of the Voronoi volume of the atoms in the vicinity of the interface. We performed this type of analysis in previous section for the justification of He trapping in Cu-Nb layered system. Now, we will expand it to the other situations.

**Figure 8:**
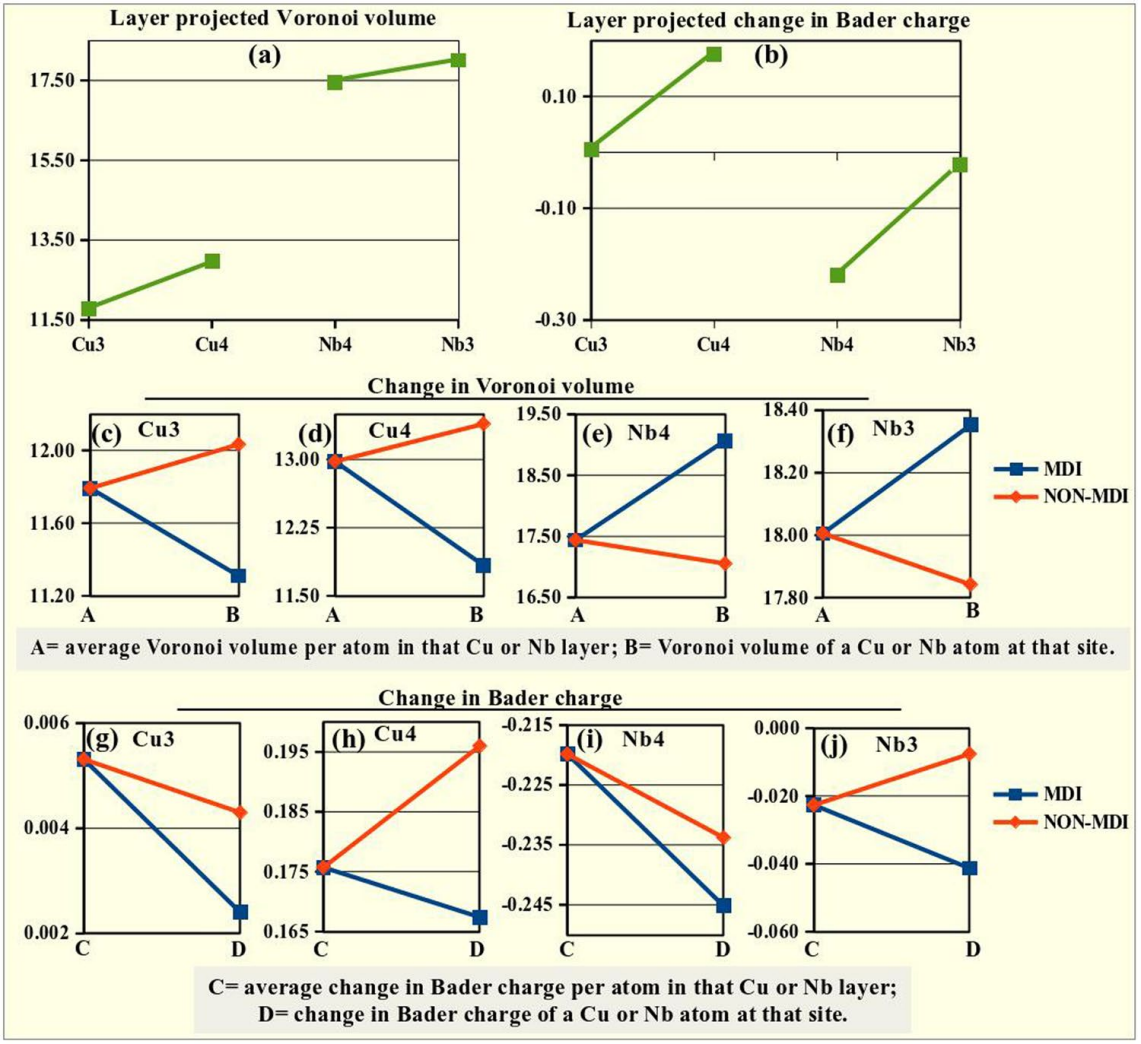
Layer projected (**a**) Voronoi volume and (**b**) change in Bader charge. The deviation from the average (for a particular layer) Voronoi volume (**c**–**f**) and change in Bader charge (**g**–**j**) at the MDI (blue line) and NON-MDI (red line) region of different layers.

Figure 8(a) shows the change in Voronoi volume as we move from the first to the second neighbouring interfacial layers. For Cu, Voronoi volume decreases as we go away from the interface, whereas the opposite is true for the Nb layers. Similar trend is followed by the layer projected change in Bader charge. The value of Bader charge increases for Cu4 and decreases for Nb4 layers (Fig. 8(b)). The Cu3 and Nb3 layers possess almost equal amount of Bader charge as their bulk counterpart. This result suggests that the metallic layers approach bulk conditions rapidly as we move away from the interface, reaffirming that a 4-layered system is sufficient for our analysis. Next, we calculate the average Voronoi volume and change in Bader charge per atom for the atomic layers in the vicinity of the interface and compare them with the respective values possessed by a metallic atom in the MDI and NON-MDI region of the individual layers. Figure 8(c–f) depicts the deviation of the Voronoi volume of a metallic atom in the MDI (blue line) and NON-MDI (orange line) region of different layers from the average Voronoi volume per atom for that layer. Same is plotted for the change in Bader charge and given in Fig. 8(g–j). In a particular Cu layer, Cu atoms in the MDI site has the lowest Voronoi volume and NON-MDI region have the highest (Fig. 8(c,d)), whereas the opposite it true for the Nb4 and Nb3 layers (Fig. 8(e,f)). From Fig. 8(g–j), we can note that the atoms in the MDI regions within a particular layer retain the lowest of excess Bader charge (blue lines).

Based on the Voronoi volume analysis we come to the conclusion that Cu4 and Cu3 experience the utmost strain and it is maximum at the MDI region of a particular atomic layer. Therefore creation of vacancy in such region will always be preferable as it will reduce the strain in that region. In fact, such trend in vacancy formation energy has been observed in the previous section of this study and also reported earlier^31^. On the other hand, Nb atoms in the Nb4 and Nb3 layers acquire more Voronoi volume compared to their bulk counterpart. Therefore, unlike in the Cu region, the creation of a vacancy in the Nb region will not release any strain energy. As a result, vacancy formation energy is almost equal in different sites of the Nb region, except at the MDI site of the Nb4 layer. At this region, the nearest neighbouring Cu atom, due to the low migration barrier, move across the interface and occupies the vacancy position. This process is accompanied by the release of a significant amount of strain energy in the Cu4 layer. Thus, this results in a low vacancy formation energy in this region. This implies that we should focus not only on the Voronoi volume of the atoms in the particular site of interest but also its nearest neighbouring atoms as well, to get a complete picture of strain energy of the system.

The correlation between He-interstitial formation energy and strain in the system through the Voronoi volume is not straight forward. Favorable site for He-interstitial formation can be identified by variation of Voronoi volume and change in Bader charge together. The change in Bader charge is nearly zero for Cu3 and Nb3 layers (Fig. 8(g,j)). Hence, the difference in He-interstitial formation energy between MDI and NON-MDI regions is very small while the magnitude is really high. In fact, the formation energy is slightly higher in the MDI regions of these layers which is an effect of the highly corrugated neighbouring regions. Atoms in the first neighbouring interfacial atomic layers possess significant variation in Bader charge, which greatly influences the He-interstitial formation energy. The change in Bader charge is positive for the Cu atoms in the Cu4 layer, implying an increase in Bader charge of the Cu atoms from their bulk counterpart. On the contrary, the negative charge in the fourth Nb layer states the decrease in Bader charge of the Nb atoms. As depicted in Fig. 8(h,i), for a particular atomic layer, Bader charge is minimum at the MDI site of that layer, where He-interstitial formation energy should also be minimum. Interestingly, this is the case for Nb4 layer, but not for the Cu4 layer. Although, this site is favourable for a He interstitial from Bader charge point of view, highly strained Cu atoms make the accommodation of a He interstial energetically expensive near it. As a result, the He atom moves towards the interfacial Nb layer in search of a favourable position. But, when we created a Cu-vacancy at the MDI site of the Cu4 layer, Bader charge at that region decreases and available Voronoi volume for He increases. Which makes the accommodation of the He atom easier. The complete picture of the Voronoi volume and change in Bader charge at each point in the interfacial Cu and Nb layers of the initial system is analysed and depicted in the SI Fig. SI7. The variation in Voronoi volume and change in Bader charge agrees well with the interpretations we have made in this section.

In this section, we have shown that with careful interpretation of Voronoi volume and change in Bader charge of the atoms in the Cu-Nb layered system, we can get a plausible qualitative picture of defect energetics in the vicinity of the interface of such large systems. This type of study should be applied to other systems in order to test its general viability for future less expensive computational analysis of the energetic mapping on defects.

## Conclusion

In summary, the formation of defects and their elemental interactions were studied in a fcc-bcc layered nanocomposite system, a viable material to be used in extreme radiation environment. Consistency of our defect formation energy with the previous DFT-based calculation is a good manifestation of convergence of the calculations. A single metallic vacancy in the interfacial Nb layer is capable of accommodating exothermically (at least) five He atoms while only one is placed in the interfacial Cu layer. On addition of more He atoms, they move closer to the Nb4 layer. Affinity of He interstitial towards metallic vacancy is explained with the help of charge density difference and Voronoi volume analysis. Nb-vacancy creates enough space and low electron density region to absorb the He interstitials. The stability of the system with He atoms in a Nb-monovacancy region was also analyzed attending the total electronic density of states at the Fermi level, leading to the highest stability when the lower value is obtained in the 5He atoms case. On the other hand, a Cu-vacancy also creates electron depleted region and available space around it, but the associated strain after insertion of the first He expels rest of them to the interfacial region. Hybridization between Cu-d and He-s states was observed, which indicates stable accommodation of He atoms in presence of a metallic vacancy. The methodology presented here based on the Voronoi volume and change in Bader charge analysis may replace the more conventional procedures used for surface energetics mapping which are extremely tedious for large systems like the Cu-Nb layered system.

## Methods

### DFT calculations

Plane-wave DFT methodology^42,43^ as implemented in the Vienna Ab-initio Simulation Package (VASP)^44,45^ was used to perform all first-principles calculations reported in this study. The Perdew-Wang 91 parametrization of the generalized-gradient approximation (GGA)^46^ was used for corrections to the non-local exchange and correlation energies and the electron-ion interactions was taken care of by projector augmented wave (PAW) pseudo potentials^47,48^. For accurate description of the electronic interaction amongst the atoms, we considered seventeen valence electrons for Cu (3*p*^6^3*d*^10^4*s*^1^), eleven for Nb (4*p*^6^4*d*^4^5*s*^1^) and two valence electrons for He (1*s*^2^2*p*^0^). A plane wave cut-off of 500 eV was set with 1E-05 eV energy convergence parameter for electronic self-consistent part. For all DFT calculations, maximum forces on every relaxed atom were converged to 0.05 eV/Å. Brillouin-Zone sampling was done using Monkhorst-Pack method with 1 × 1 × 1 k-point mesh for ionic relaxation and 4 × 4 × 1 k-point mesh for density of states (DOS) calculations. Bader charge analysis was performed with the code written by Henkelman’s group^49–52^ using the default cutoff values of atomic radii (covalent radius of each element) for each element recommended by VASP.

To model the Cu-Nb layered system, four layers of fcc 〈111〉 planes of Cu and four layers of bcc 〈110〉 planes of Nb were joined according to the KS orientation relationship^27,28^. To construct these layers, the lattice parameters of individual Cu and Nb layers were fixed to their bulk values, i.e., 3.615 Å for Cu and 3.30 Å for Nb. Following Metsanurk *et al*. work^29^, a quasi unit cell was constructed; the lattice vectors of this unit-cell being 23.00 Å along X-direction and 13.30 Å along Y-direction, connecting the nearest MDI sites along the respective directions. With these lattice parameters, each Cu layer consists of 54 Cu atoms (9 × 6) and each Nb layer consists of 40 Nb atoms (8 × 5). The Z-direction was defined by the total number of Cu and Nb layers including the interface. To avoid interactions between periodic images of the supercell a vacuum spacing of 16 Å was added along Z-direction. The starting separation between the two metals was fixed at 2.33 Å (closest to the distance between two Nb 〈110〉 planes). Thus, the final separation is optimized by minimizing the energy of the system during the structural relaxation (see SI, Fig. SI1).

### Energy calculation

Having obtained the total energies under different defect-host conditions from DFT calculations, analytical expressions were used to calculate the formation energies. For various energy calculations, we used the term ${E}_{c}({{\rm{\Lambda }}}_{conf}^{Atoms})$, where *E*_*c*_ denotes the total energy of the system with configuration ${{\rm{\Lambda }}}_{conf}^{Atoms}$. The acronym *conf* refers to the type of defect in the system and *Atoms* specify the type of atoms used in the calculation. These notations are similar to the one used by T. Ohnuma *et al*.^17^ for defect study in bcc Fe. Applying this notation, we can define the formation energy of each defect as,

1 $${E}_{f}={E}_{c}({{\rm{\Lambda }}}_{conf}^{Atoms})+{n}_{vac}{E}_{metal}-n{E}_{He}-{E}_{c}({{\rm{\Lambda }}}^{Cu-Nb})$$

The notations, ${E}_{c}({{\rm{\Lambda }}}_{V}^{Cu-Nb})$, ${E}_{c}({{\rm{\Lambda }}}_{nHe}^{Cu-Nb,He})$ and ${E}_{c}({{\rm{\Lambda }}}_{V+nHe}^{Cu-Nb,He})$, represents the energies of the relaxed systems containing a monovacancy (*V*), n He-interstitial atoms and a *V* + *nHe* complex respectively. *n*_*vac*_ is the number of vacancy and *n* is the number of He interstitial, *E*_*metal*_ is the energy of a Cu or Nb atom in their bulk configuration and *E*_*He*_ is the energy of an isolated He atom placed inside a large empty simulation box. *E*_*c*_(Λ^*Cu*−*Nb*^) is the energy of the initial interface.

We defined the trapping energy *E*^*trap*^ to characterize the energy required for moving a He atom into the metallic monovacancy from a distant interstitial site. For *n* He atom trapped in the metallic monovacancy the trapping energy is obtained as:

2 $${E}^{trap}(nHe)={E}_{c}({{\rm{\Lambda }}}_{V+nHe}^{Cu-Nb,He})-{E}_{c}({{\rm{\Lambda }}}_{V+(n-\mathrm{1)}He}^{Cu-Nb,He})-{E}_{c}({{\rm{\Lambda }}}_{V+H{e}_{dist}}^{Cu-Nb,He})+{E}_{c}({{\rm{\Lambda }}}_{V}^{Cu-Nb})$$

Here, ${E}_{c}({{\rm{\Lambda }}}_{V+nHe}^{Cu-Nb,He})$ and ${E}_{c}({{\rm{\Lambda }}}_{V+(n-\mathrm{1)}He}^{Cu-Nb,He})$ are the energies of the supercell with a monovacancy plus *n* and (*n* − 1) *He* atoms, respectively; ${E}_{c}({{\rm{\Lambda }}}_{V+H{e}_{dist}}^{Cu-Nb,He})$ is the energy of the supercell with a metallic monovacancy and an interstitial He atom far away from the monovacancy site. We choose distant interstitial site for He-interstitial in the Cu2 (Nb2) layer at a distance >10 Å from the metallic vacancy at the MDI site of the Cu4 (Nb4) layer. ${E}_{c}({{\rm{\Lambda }}}_{V}^{Cu-Nb})$ is the energy of the supercell with a vacancy.

The total binding energy of *V* + *nHe* complex was defined as,

3 $${E}_{b}^{T}(V+nHe)=\{{E}_{c}({{\rm{\Lambda }}}_{V}^{Cu-Nb})+n{E}_{c}({{\rm{\Lambda }}}_{He}^{Cu-Nb,He})\}-\{n{E}_{c}({{\rm{\Lambda }}}^{Cu-Nb})+{E}_{c}({{\rm{\Lambda }}}_{V+nHe}^{Cu-Nb,He})\}$$

where, *n* is the number of He interstitial taken into consideration.

Using the above definition we can calculate the binding energy between a *He* atom and a *V* + (*n* − 1)*He* complex to form a *V* + *nHe* complex as,

4 $${E}_{b}(V+nHe)={E}_{b}^{T}(V+nHe)-{E}_{b}^{T}(V+(n-\mathrm{1)}He)$$

## Electronic supplementary material

Supplementary information
