# Supplementary material for: Vacancy assisted He-interstitial clustering and their elemental interaction at fcc-bcc semicoherent metallic interface
Source: Sci Rep. 2018 Mar 1;8:3844. doi: 10.1038/s41598-018-22141-y (PMC5832800; doi:10.1038/s41598-018-22141-y)
Supplement: Supplementary file 1 — Supplementary information [file 41598_2018_22141_MOESM1_ESM.pdf]

Supporting Information for:

Vacancy assisted He-interstitial clustering and their elemental interaction at fcc-bcc semicoherent metallic interface

Ujjal Saikia,[1] Munima B. Sahariah,[1,*] Cesar González[2] and Ravindra Pandey[3]

[1]Institute of Advanced Study in Science and Technology, Guwahati 781035, India

[2]Departamento de Física Teóica de la Materia Condensada and Condensed Matter Physics Center (IFIMAC), Facultad de Ciencias. Universidad Autónoma de Madrid, E-28049 Madrid, Spain

[3]Department of Physics, Michigan Technological University, Houghton, MI 49931-1295, USA

AUTHOR INFORMATION

Corresponding Author

*E-mail to: munima@iasst.gov.in

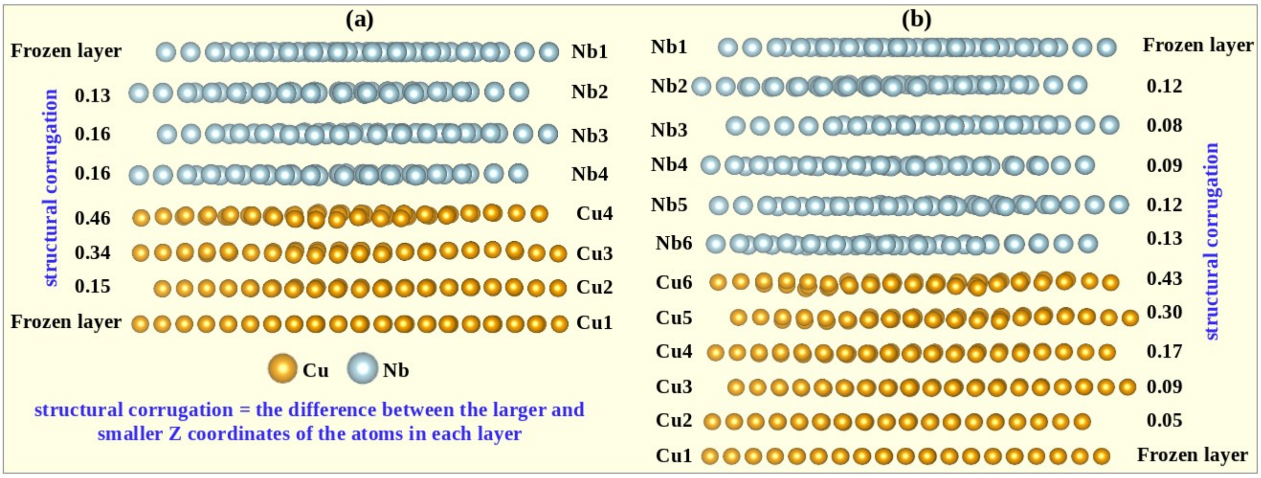

Figure SI1: A visual comparison of (a) the 4-layered structure with (b) the 6-layered structure. The value near each atomic layer is the amount of structural corrugation in that layer (in Å unit). Throughout this study we have used same notation as given here (Cu1, Nb1,....etc.) to identify the atomic layers.

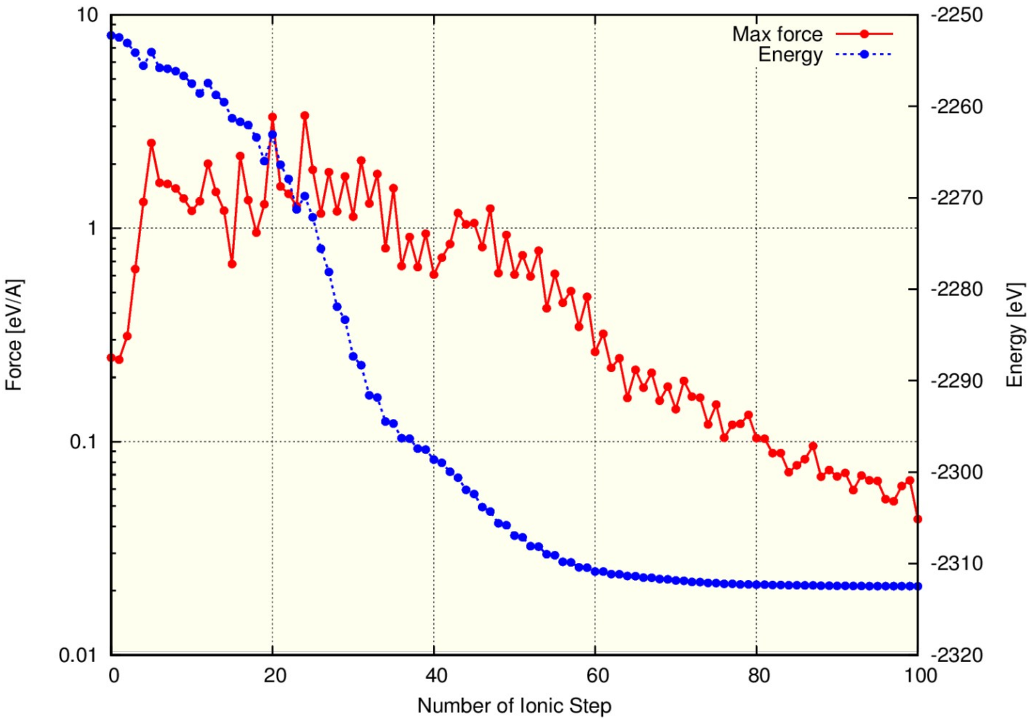

Figure SI2: Variation in total energy (blue line) and force per atom (red line) of the 4-layered system during ionic relaxation.

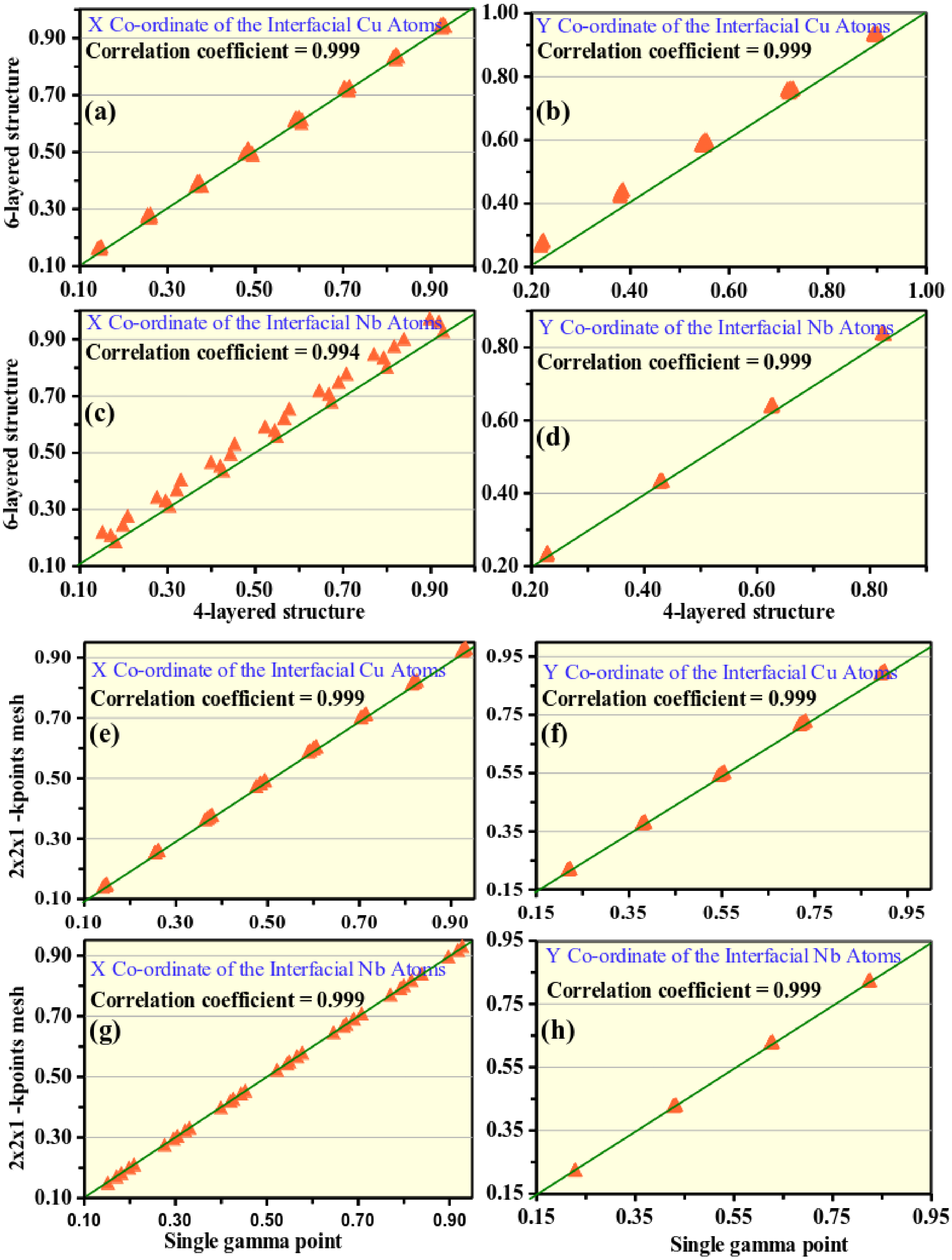

Figure SI3: Scattering plot for x and y co-ordinates of the interfacial Cu and Nb atoms.

The size of the supercell used in DFT simulation should be large enough to emulate the interface topology of the semicoherent interface as well as to capture the original strain field generated near the interface due to lattice point mismatch at the interface. Two different first-principle approaches have been made previously, one with 4-layered Cu/3-layered Nb [ref. SI1] and another with 6-layered Cu/6-layered Nb [ref. SI2] to mimic the Cu-Nb layered nanocomposite system. The 6-layered system with 9 kpoints apprehend the interface property very well but, it requires 324 Cu and 240 Nb atoms which is computationally very expensive. In the 3-layered system, they have not considered any fixed outer layer to mimic the bulk properties in the system.

The structural corrugation (the difference between the larger and smaller Z coordinates of the atoms in each layer) can be considered as the adequate parameter to validate the size of the simulation cell because it measures the strain field in terms of structural deformation in the system. We obtained the same trend for 4-layered and 6-layered structures. For the interfacial Cu layers, corrugation decreases rapidly (from 0.46 Å to a much smaller value of 0.15 Å for the third Cu layer) as shown in supporting information (SI) Fig. SI1. On the other hand, corrugation for the interfacial Nb layer is very small (0.16 Å) in comparison to the equivalent Cu layer and it remains almost stable (0.13 Å) in the subsequent layers (Fig. SI1), implying that a 4-layered structure is sufficient to capture the strain field generated near the interface.

The layered system used in this study consisting of four layers of Cu and four layers of Nb is well optimized with respect to the total energy and force per atom in the system (convergence plot for the initial system during ionic relaxation is given in Fig. SI2). The effect of layer number and k-points considered in this study on the arrangement of the atoms at the interface was also investigated by examining the displacement of the interfacial atoms as shown in Fig. SI3. The points in the plots represent the X and Y coordinates of the interfacial metal atoms and the straight lines are the lines of correlation. The plot for the 4-layered structure against 6-layered structure as given in Fig. SI3 (a)-(d) implies that the displacements of the interfacial atoms are similar within these two structures (if they were identical, the points in the plot would lie on the straight line) having correlation coefficient > 0.99. Hence, we can conclude that the 4-layered and 6-layered structures give us the same interface topology for the layered system. A similar trend in structural corrugation is obtained along Z-direction in the 4-layered structure with a single Γ-point as well as with a $2\times2\times1$ k-mesh. The X and Y coordinate plot of the interfacial Cu and Nb atoms in the 4-layered structure with single Γ-point against $2\times2\times1$ k-mesh (Fig. SI3(e)-(h)) show that both k-space samplings lead us to similar interface topology.

Table SI1: V, He and VHe complex formation energies calculated for their stable configurations for their stable configurations at the MDI region of the first neighboring interfacial layer. Energies are in eV unit.

System	Monovacancy (V)		He-interstitial		VHe-complex	
	Cu layer	Nb layer	Cu layer	Nb layer	Cu layer	Nb layer
4-layered (calculated) (GGA-PW91)	0.20	1.15	2.48	2.48	1.98	2.95
4-layered (calculated) (GGA-PBE)	0.29	1.25	2.52	2.52	2.08	-
6-layered (ref. SI2) (GGA-PBE)	0.32	1.29	2.50	2.50	2.07	2.73

The values of formation energy reported in reference [ref. SI2] are slightly different from the values we have reported in this work. This difference in monovacancy formation energy arises dominantly because of the different exchange-correlation functionals. As mentioned in the "Methods- DFT calculations" section, we have used Perdew-Wang 91 (PW91) parametrization of the generalized-gradient approximation (GGA). Whereas, the 6-layered study performed by C. González *et al.* [ref. SI2] used Perdew-Burke-Ernzerhof (PBE) parametrization of the generalized-gradient approximation. For proper comparison, we have therefore calculated the V, He-interstitial and VHe complex formation energies at the MDI site of the first neighboring interfacial Cu and Nb layers for the 4-layered system with PBE parametrized GGA functional also. For PW91 functional, the percentage difference in defect formation energy values between this work and ref. SI2 is in the range of 46.15% - 1.99% which reduces to 9.82% - 0.96%, when we used PBE parametrized GGA functional. Comparative defect energetic values for all the three cases are listed in Table SI1 above.

Table SI2: Corrugation in different layers for the initial system and the systems with various defect configurations at the MDI region of the 1st neighboring interfacial Cu and Nb layer. The values are in Å unit.

#	Initial	V+nHe at the MDI region of Cu4 layer					V+nHe at the MDI region of Nb4 layer				
		V+1He	V+2He	V+3He	V+4He	V+5He	V+1He	V+2He	V+3He	V+4He	V+5He
Nb1	0.00	0.00	0.00	0.00	0.00	0.00	0.00	0.00	0.00	0.00	0.00
Nb2	0.13	0.13	0.13	0.13	0.13	0.13	0.14	0.14	0.13	0.14	0.14
Nb3	0.16	0.18	0.16	0.13	0.12	0.22	0.15	0.14	0.12	0.13	0.15
Nb4	0.16	0.23	0.13	0.27	0.51	0.82	0.14	0.13	0.15	0.16	0.20
Cu4	0.46	0.41	0.51	0.50	0.51	0.59	0.37	0.39	0.45	0.50	0.51
Cu3	0.34	0.28	0.32	0.28	0.32	0.35	0.27	0.28	0.29	0.32	0.32
Cu2	0.15	0.14	0.16	0.15	0.16	0.18	0.15	0.15	0.15	0.15	0.15
Cu1	0.00	0.00	0.00	0.00	0.00	0.00	0.00	0.00	0.00	0.00	0.00

No previous data is available for VHe complexes with more than one He. So, to ensure that the behavior of the point defects is not affected by the number of layers taken into consideration as we go on adding more He atoms, we have examined the deformation of the system for various defect clusters. To that end, we have calculated the corrugation in various Cu and Nb layers for different defect complexes considered in this study. As we have tabulated in table SI2 the corrugation in the 1st and 2nd neighboring interfacial layers (Cu3, Cu4, Nb3 and Nb4) increases as we increase the size of the defect complex. But we haven't observed any significant change in corrugation in the 3rd neighboring interfacial layers (Cu2 and Nb2, highlighted rows in table SI2). This implies that the strain field generated by the defect complexes is primarily limited to the 1st and 2nd neighboring interfacial layers. Hence, we insist that the 4-layered structure is sufficient to capture the strain field generated by up to (at least) V+5He complex considered in this study.

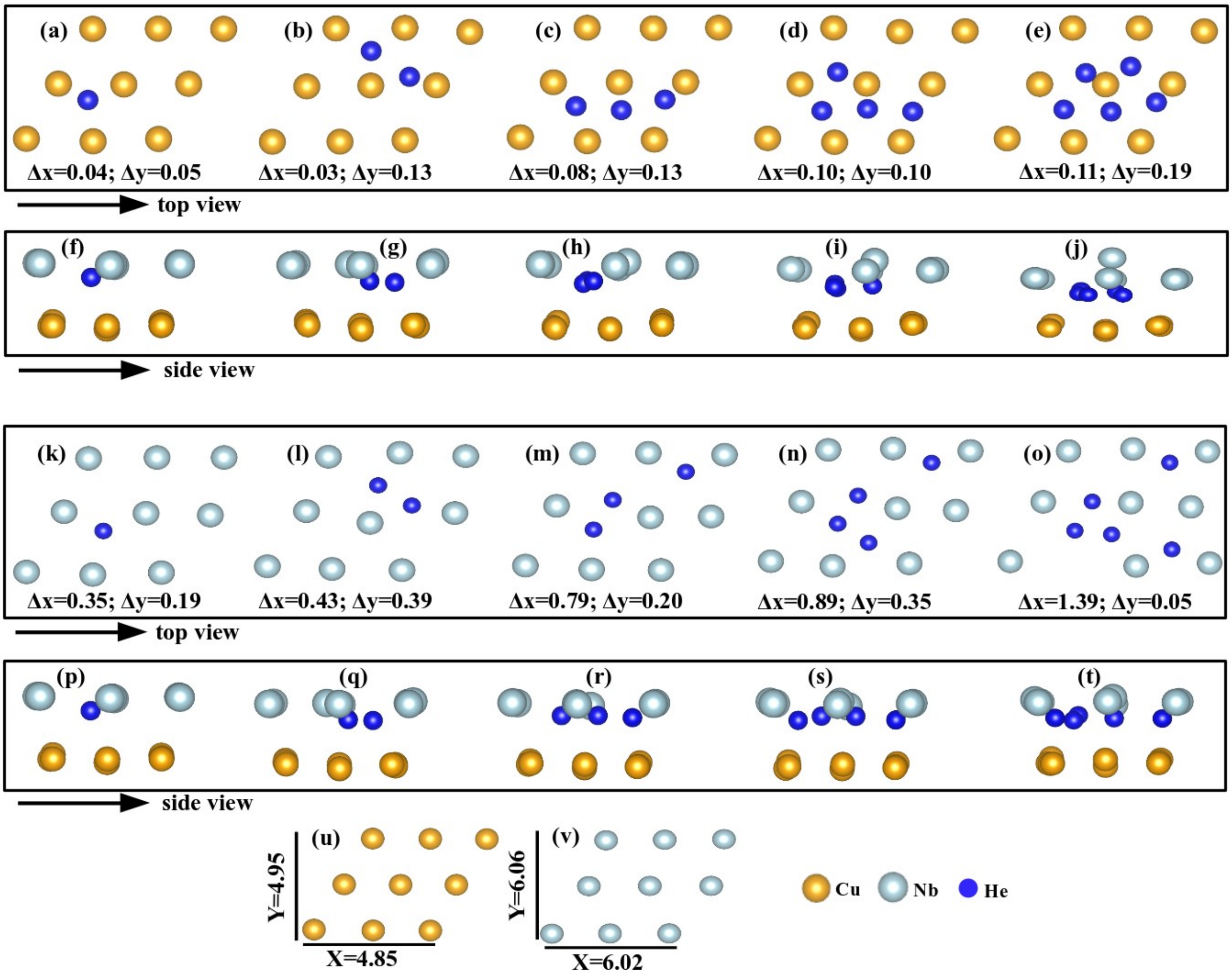

Figure SI4: Optimized structure of the nHe (n=1,2,3,4 and 5) complexes inserted at the MDI region of the interfacial Cu (Fig. (a) to (e)) and Nb layers (Fig. (k) to (o)). Figures (u) and (v) show MDI region for the initial interfacial Cu and Nb atomic planes. The x and y values represent the horizontal and vertical dimensions of the respective sites in Å unit. Δx and Δy given at the bottom of the figures represent the change in dimension of the MDI region after insertion of the He atoms. The positive values symbolize the increase in the dimension of the respective MDI region.

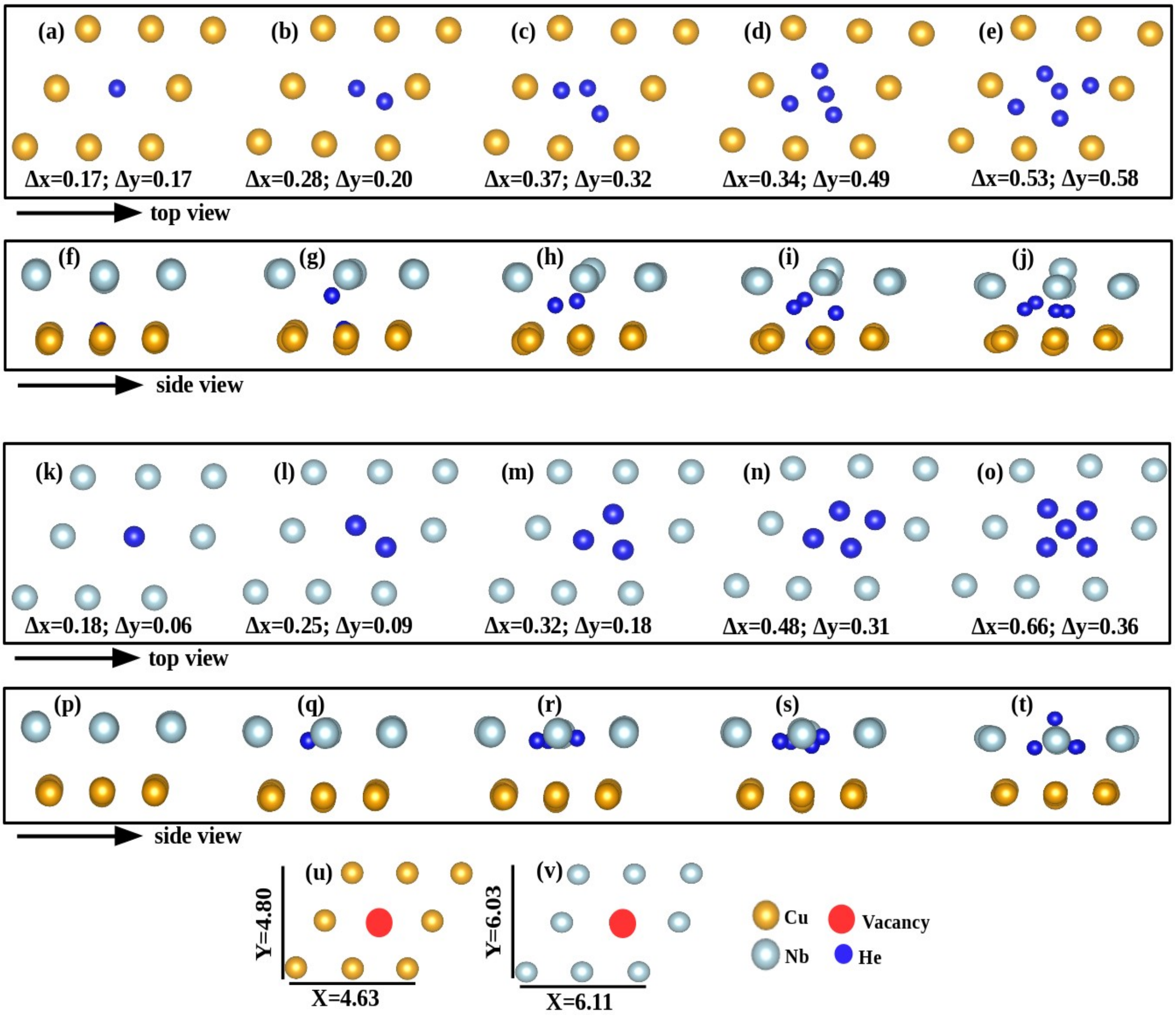

Figure SI5: Optimized structure of the V+nHe (n=1,2,3,4 and 5) complexes inserted at the MDI region of the interfacial Cu (Fig. (a) to (e)) and Nb layers (Fig. (k) to (o)). Figures (u) and (v) show the MDI region for the defective interfacial Cu and Nb atomic planes. The x and y values represent the horizontal and vertical dimension of the respective sites in Å unit. Δx and Δy given at the bottom of the figures represent the change in dimension of the MDI region after insertion of the He atoms. The positive values symbolize the increase in the dimension of the respective MDI region.

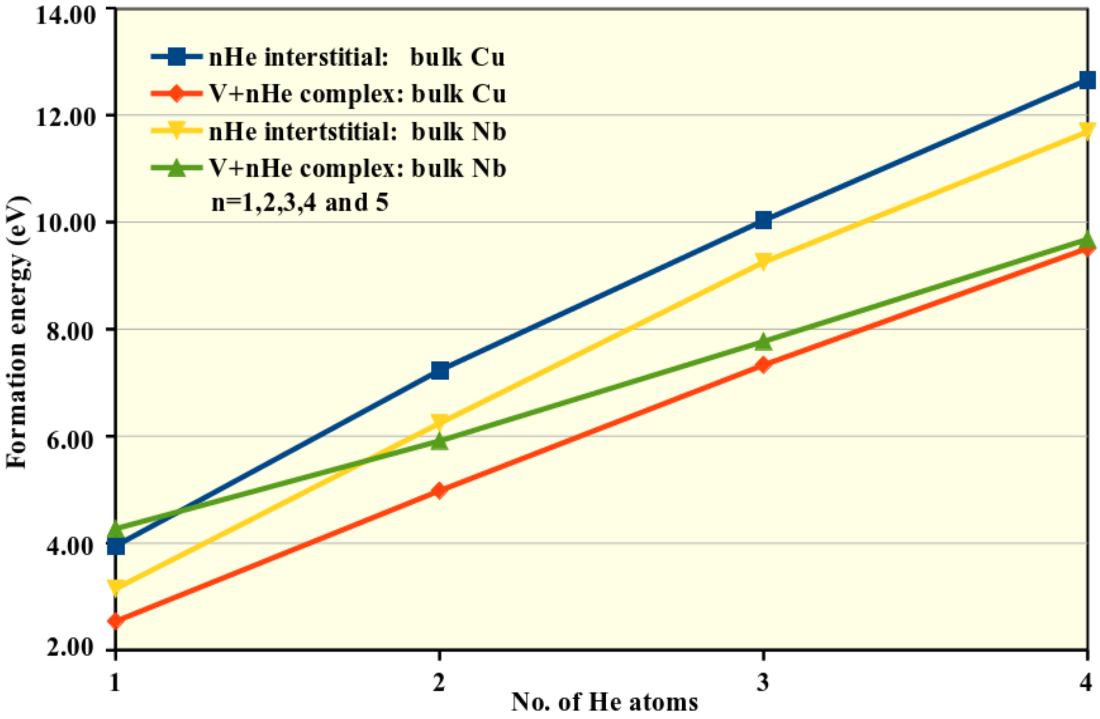

Figure SI6: Formation energies of nHe and V+nHe complexes in bulk Cu and Nb. Values are taken from references SI3 and SI4.

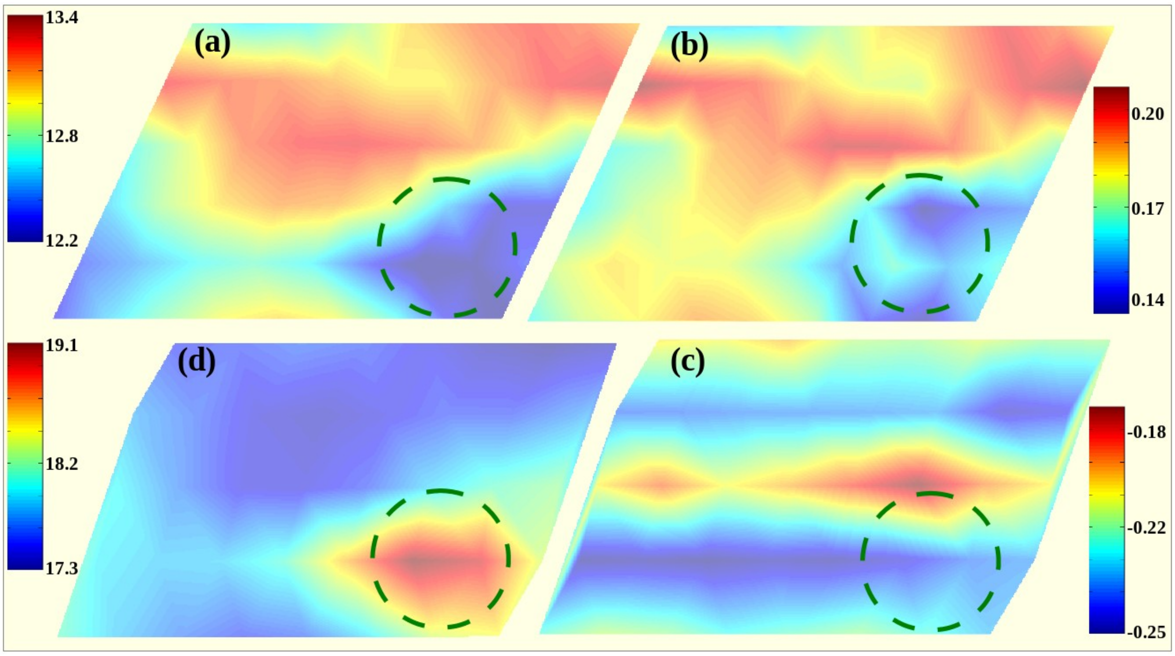

Figure SI7: The distribution of (a) Voronoi volume ($Å^3$) and (b) change in Bader charge (|e|) for the interfacial Cu layers. The same is depicted for the interfacial Nb layer in (c) and (d). The MDI region is indicated by the dashed circle.

Table SI3: Formation energies of V, nHe and V+nHe (n=1,2,3,4 and 5) complexes at Cu-Nb layered systems and their respective bulk counterparts in eV.

Type of defect	Interfacial (this work)		Bulk	
	Cu plane	Nb plane	Cu [ref. SI3]	Nb [ref. SI4]
V	0.20	1.15	1.08	2.67
1He	2.48	2.48	3.95	3.15
2He	5.29	5.28	7.23	6.24
3He	7.63	7.92	10.03	9.25
4He	10.35	10.60	12.66	11.69
5He	12.44	13.49	-	-
V+1He	1.98	2.95	2.54	4.27
V+2He	4.28	4.42	4.98	5.91
V+3He	6.599	5.79	7.33	7.77
V+4He	8.779	7.55	9.51	9.68
V+5He	10.99	9.22	-	-

Table SI4: Charge transfer from the metallic matrix to the He atoms for the V+nHe (n=1,2,3,4 and 5) complexes at the interfacial MDI region of the Cu and Nb layers.

Complexes	# He	Amount of charge transfer (|e|)	
		MDI: Cu4	MDI: Nb4
V+1He	1	0.105	0.099
V+2He	1	0.098	0.090
	2	0.130	0.100
V+3He	1	0.110	0.087
	2	0.105	0.083
	3	0.084	0.092
V+4He	1	0.074	0.076
	2	0.113	0.091
	3	0.098	0.094
	4	0.078	0.076
V+5He	1	0.066	0.090
	2	0.069	0.074
	3	0.113	0.086
	4	0.095	0.080
	5	0.087	0.082

Supporting Information References

[SI1] Metsanurk, E., Tamm, A., Caro, A., Aabloo, A. & Klintenberg, M. First-principles study of point defects at a semicoherent interface. *Sci. Reports* **4**, 7567–7570 (2014).

[SI2] González, C., Iglesias, R. & Demkowicz, M. J. Point defect stability in a semicoherent metal lic interface. *Phys. Rev. B* **91**, 064103–064109 (2015).

[SI3] González, C., Fernandez-Pello, D., Cerdeira, M. A., Palacios, S. L. & Iglesias, R. Helium bubble clustering in copper from first principles. *Model. Simul. Mater. Sci. Eng.* **22**, 035019–035035 (2014).

[SI4] Cerdeira, M., Palacios, S., González,, C., Fernandez-Pello, D. & Iglesias, R. Ab initio simu lations of the structure, energetics and mobility of radiation-induced point defects in bcc Nb. *J. Nucl. Mater.* **478,** 185–196 (2016).
